# Single Incision Cholecystectomies for Acute Cholecystitis: A Single Surgeon Series from the Caribbean

**DOI:** 10.1155/2022/6781544

**Published:** 2022-02-16

**Authors:** Shamir O. Cawich, Sanjib K. Mohanty, Omar Felix, Giovanni Dapri

**Affiliations:** ^1^Department of Surgery, University of the West Indies, St. Augustine Campus, Trinidad, Trinidad and Tobago; ^2^Department of Surgery, Cayman Islands Hospital, George Town, Cayman Islands; ^3^Department of Gastrointestinal Surgery, European School of Laparoscopic Surgery, Saint-Pierre University Hospital, Brussels, Belgium

## Abstract

**Introduction:**

Single incision laparoscopic surgery (SILS) is accepted as a safe alternative to conventional multiport laparoscopic (MPL) cholecystectomy for benign gallbladder disease. Since many surgeons carefully select patients without inflammation, there are limited data on SILS for acute cholecystitis. We report a single surgeon experience with SILS cholecystectomy for patients with acute cholecystitis.

**Materials and Methods:**

After securing ethical approval, we performed an audit of all SILS cholecystectomies for acute cholecystitis by a single surgeon from January 1, 2009, to December 31, 2019. The following data were extracted: patient demographics, intraoperative details, surgical techniques, specialized equipment utilized, conversions (additional port placement), morbidity, and mortality. Data were analyzed using SPSS 12.0.

**Results:**

SILS cholecystectomy was performed in 25 females at a mean age of 35 ± 4.1 (SD) years and a mean BMI of 31.9 ± 3.8 (SD) using a direct fascial puncture technique without access platforms. The operations were completed in 83 ± 29.4 minutes (mean ± SD) with an estimated blood loss of 76.9 ± 105 (mean + SD). Three (12%) patients required additional 5 mm port placement (conversions), but no open operations were performed. The patients were hospitalized for 1.96 ± 0.9 days (mean ± SD). There were 2 complications: postoperative superficial SSI (grade I) and a diaphragmatic laceration (grade III). No bile duct injuries were reported. There were 9 patients with complicated acute cholecystitis, and this sub-group had longer mean operating times (109.2 ± 27.3 minutes) and mean postoperative hospital stay (1.3 ± 0.87 days).

**Conclusion:**

The SILS technique is a feasible and safe approach to perform cholecystectomy for acute cholecystitis. We advocate a low threshold to place additional ports to assist with difficult dissections for patient safety.

## 1. Introduction

Navarra et al. were the first to perform cholecystectomy via single incision laparoscopy (SIL) in 1997 [1]. It is now accepted as a feasible and safe option to conventional laparoscopy (CL) with multiple ports for gallbladder disease in the twenty-first century [2–9]. Most authors will agree that SIL is technically difficult due to the frequency of instrument collision, counterintuitive movements, and tunneled vision. Therefore, most surgeons carefully select patients without inflammation, and there are limited data on SIL for acute cholecystitis. We report our experience with emergency SIL cholecystectomy for patients with acute cholecystitis.

## 2. Methods

After securing ethical approval (ECP 115/2011/12) from the review board, we performed an audit of a prospectively maintained database of patients who underwent cholecystectomy by a single surgeon from January 1, 2009, to December 31, 2019. We excluded patients who had open cholecystectomy, CL cholecystectomy, and elective operations. The inclusion criteria were patients with ultrasound-confirmed acute cholecystitis who had emergency cholecystectomy during the same admission for acute cholecystitis that were completed via SIL.

The diagnosis of acute cholecystitis was made when there were suggestive clinical findings and at least 2 of these sonographic findings: (1) distended gallbladder >50 mls; (2) pericholecystic fluid, (3) wall thickness ≥5 mm, and/or (4) a sonographic Murphy's sign. In this series, all patients with acute cholecystitis were booked for emergent operations during the same admission, as the operating schedule allowed.

We utilized the standardized definition of complicated acute cholecystitis proposed by Meekin et al. [10]: operative or histopathologic findings of gallbladder ulceration, fibrous exudation, necrosis, perforation, and/or empyema. These patients were anticipated to have technically difficult operations compared to those with uncomplicated cholecystitis who had only gallbladder distention, mural oedema, and transmural cellular infiltration. A SIL cholecystectomy was defined as one in which a single umbilical incision was created to pass all laparoscopic instruments. A conversion was defined as the need for additional incisions for extra ports.

The clinical records for all patients who had emergency SIL cholecystectomy for acute cholecystitis were retrieved, and the following data were extracted: patient demographics, intraoperative details, surgical techniques, specialized equipment utilized, conversions, morbidity, and mortality. Data were analyzed using SPSS 12.0.

## 3. Results

Over the study period, SIL cholecystectomy was performed for acute cholecystitis in 25 females at the age of 35 ± 4.1 years (mean ± SD) and a BMI of 31.9 ± 3.8 (mean ± SD). *The absence of male patients in this study, while interesting, was purely coincidental.* All patients who met an indication for cholecystectomy were counseled by the attending surgeon and given a choice to select the approach.

**Figure 1:**
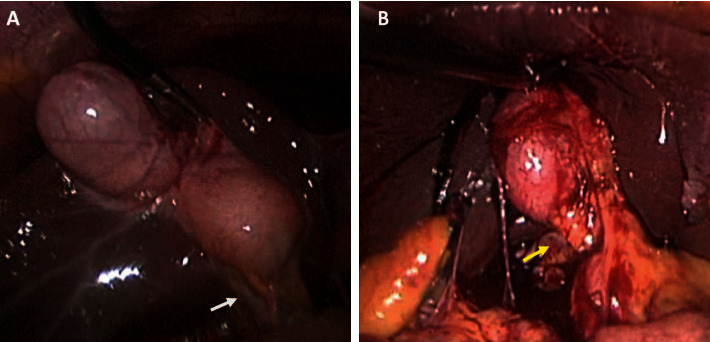
A comparison of the intraoperative views during SILS cholecystectomy in a patient without acute inflammation (a) and one with acute cholecystitis (b). The mural erythema and oedema in the patient with acute cholecystitis make grasping the gallbladder difficult and limit exposure of Calot's triangle (arrows).

**Figure 2:**
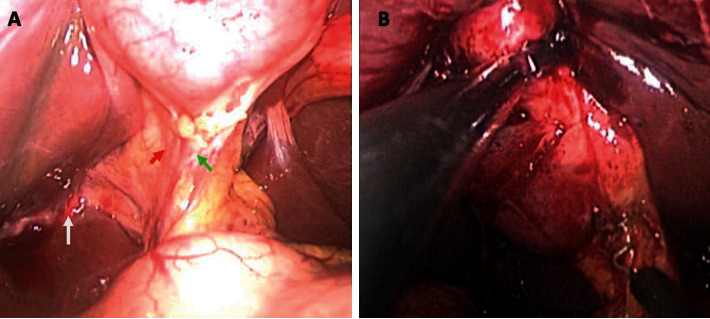
(a) Intraoperative views of structures in Calot's triangle during SILS cholecystectomy in a patient without inflammation and (b) one with acute cholecystitis. In (a), the cystic artery (red arrow), cystic duct (green arrow), and Rouviere's sulcus (white arrow) are visible across the thin, uninflamed peritoneum even before dissection, but the structures are not readily visible in the patient with acute cholecystitis.

Access to the peritoneal cavity was always achieved with an open technique at the umbilicus to establish a 12 mmHg pneumoperitoneum. In all cases, we used a *standard 30-degree laparoscope* and a previously described direct fascial puncture technique without special access platforms [11]. Standard operative techniques were used for intracorporal dissection. Compared to elective operations, we found that there were two steps at which we experienced difficulty in patients with acute cholecystitis: effectively grasping the gallbladder to provide good views (Figure 1) and dissecting the structures in Calot's triangle (Figure 2). However, controlled and patient dissection allowed us to identify Strasberg's critical view *of safety* in all cases. Once the cystic duct and artery were secured, the gallbladder was separated from the liver bed with electrocautery.

The operations were completed in 83 ± 29.4 minutes (mean ± SD) with an estimated blood loss of 76.9 ± 105 mls (mean + SD). In 3 cases, an additional 5 mm port was placed to assist with the dissection. This was considered a SILS conversion (12%), but no conversions to open surgery were required. The incision length was 16.4 mm ± 3.5 mm (mean + SD). The patients were hospitalized for 1.96 ± 0.9 days (mean ± SD).

There were 2 complications recorded: postoperative superficial surgical site infection (grade I) and a diaphragmatic laceration during cautery dissection of dense perihepatic adhesions (grade III). One additional 5 mm port was placed to for suture repair of the diaphragm. There were no bile duct injuries reported.

There were 9 **(36%)** patients with complicated acute cholecystitis: gallbladder empyema (5), gangrenous cholecystitis (2), and gallbladder perforations (2). In the subgroup of acute complicated cholecystitis, mean operating time was longer at *109.2 ± 27.3 minutes* (range 70–154, median 106). All three conversions (addition of an extra port to assist dissection) and both complications occurred in this subgroup, but there was no mortality or major morbidity. The mean postoperative hospital stay in this group was 1.3 days (range 1–4, median 2, SD + 0.87).

## 4. Discussion

SIL cholecystectomy is performed in an attempt to further reduce the invasive nature of CL. It is currently accepted as a safe alternative to CL. To date, there have been six published randomized controlled trials comparing SIL and CL [2–7]. These trials showed that SIL cholecystectomy was associated with significantly greater patient satisfaction [2, 7], improved quality of life [3], and better cosmesis [2–7]. Two subsequent meta-analyses of the studies comparing SIL and CL cholecystectomy have been published [8, 9]. Markar et al. [8] demonstrated that both techniques had equivalent morbidity, postoperative pain scores, and duration of hospitalization. Evers et al. [9] demonstrated that both techniques had similar mortality and duration of hospital stay, but SIL was accompanied by increased morbidity, better pain scores, and superior cosmesis. The available data do not demonstrate superiority for SIL, but shows that SIL is a safe alternative to CL cholecystectomy.

Most surgeons will agree that SIL cholecystectomy is a technically challenging procedure due to frequent instrument clashes, vision in-line with working instruments, and counterintuitive hand movements. Therefore, many reports in the literature document the outcomes with SIL for elective cholecystectomy in the absence of acute inflammation or in small numbers of patients with acute cholecystitis mixed among larger study populations. We found two published reports that specifically detailed outcomes in patients with acute cholecystitis [12, 13].

Ikumoto et al. [12] examined 100 patients who underwent SIL cholecystectomy for acute cholecystitis and reported 87.4 minutes of mean operating time, 80.6 mls of mean estimated blood loss, 12% conversions, 4% major morbidity, and 5.7 days postoperative hospital stay. They were able to achieve the critical view in 89% of patients. Chuang et al. [13] compared the surgical outcomes after cholecystectomy in 108 patients with acute cholecystitis using the SIL (62) versus CL (46) approaches. They reported that the techniques brought similar operative time, estimated blood loss, postoperative pain, conversions, and morbidity, but SIL resulted in significantly reduced postoperative hospitalization.

In our experience, operating time (83 mins) compared favorably to reports in the literature that ranged from 87.4 [12] to 101 [13] minutes. As expected, the mean operating time was longer in the subgroup of patients with complicated acute cholecystitis (109.2 ± 27.3 minutes). Only the report by Chuang et al. [13] specifically detailed operating time in patients with complicated acute cholecystitis (119.8 ± 38.8 mins), and it compared favorably to that in our series.

Using the direct fascial puncture technique without working ports, we found a good combination of dexterity and reduced instrument clashes. This was discussed in detail in a prior report [11], but we believe it contributed to the low rate of conversion in our series. Our conversion rates (12%) were comparable to those in the published literature for patients with acute cholecystitis that ranged from 12% [12] to 24% [13].

There were two complications in this series. Generally, the morbidity rate was comparable to reports in the medical literature that range from 4% [12] to 9.7% [13]. Both of our complications occurred in the subgroup of patients with complicated acute cholecystitis. Chuang et al. [13] reported complications in 4.3% of patients with complicated acute cholecystitis (subhepatic abscess requiring drainage). Ikumoto et al. [12] reported 4% major morbidity in patients with complicated acute cholecystitis: bile leaks (2) and stone passage into the common duct (2).

Many surgeons are reluctant to attempt laparoscopic cholecystectomy for acute cholecystitis because of the perceived technical difficulty that results from handling friable inflamed tissues, bleeding while dissecting adhesions, and obscured vision due to phlegmon formation. This has already been thoroughly investigated in *meta-analyses* of prospective randomized trials in patients undergoing CL for acute cholecystitis [14–18]. A Cochrane systematic review [14] that compared early and delayed multiport cholecystectomy for acute cholecystitis in 488 patients demonstrated similar bile duct injury rates (0.4% vs. 0.9%), mortality (0), serious morbidity (6.5% vs. 5.0%), and conversions (19.7% vs. 22.1%). Caribbean data are similar, with no statistical difference in overall morbidity, major morbidity, bile duct injury, or mortality between the groups [19, 20].

Our results with SIL cholecystectomy are comparable to reports from the region when CL cholecystectomy is performed for acute cholecystitis in which overall morbidity ranges from 6.2% [19] to 8.1% [20], operating time ranges from 86 [19] to 99 minutes [20], blood loss ranges from 79 [19] to 93 mls [20], and conversions from 0 [19] to 4.1% [20].

We acknowledge that the SIL technique is challenging due to reduced triangulation, instrument collision, and a view with the laparoscope parallel to working instruments. Therefore, surgeons with advanced laparoscopic skills should only use this approach in patients with acute cholecystitis after gaining considerable experience with SIL for elective cholecystectomies. Additionally, there should be a low threshold to place an additional port in difficult cases, converting to CL to ensure patient safety.

This approach is not usually used in patients with acute cholecystitis. This explains the small number of patients in this study. Nevertheless, we acknowledge that this study contains small patient numbers and this is a limitation of the study methodology. There should be further studies auditing larger number of patients undergoing SIL for acute cholecystitis or randomized controlled trials comparing SIL and CL.

## 5. Conclusions

The SILS technique is a feasible and safe alternative to conventional MPL cholecystectomy for acute cholecystitis. There should be a low threshold to place additional ports to convert to conventional laparoscopy.

## Data Availability

The raw data in this study can be accessed from the corresponding author upon request.
